# Congestive Fever

**Published:** 1843-06

**Authors:** M. M. Pallen

**Affiliations:** St. Louis, Mo.


### Art. IV.—*Congestive Fever.* By M. M. Pallen, M. D. of St. Louis, Mo.

Amongst the various forms of fever which prevail in the south-western part of the United States, there is none more entitled to the investigation of the pathologist than that which has been called Congestive Fever. The frequency of its appearance, and the ravages it commits, seriously demand that it should be studied with a view to ascertain its true nature, so as to enable us to meet it promptly, energetically, and skilfully. The investigation of disease requires something more than a mere observance of symptoms; it is necessary to trace those symptoms to their causes, to understand the pathology of morbid actions, and to apply judiciously our therapeutical indications.

Impressed with this view, the writer of this essay endeavored, during a residence of seven years in Vicksburg, Mississippi, to embrace the many opportunities he had of studying the nature of this formidable malady, and to ascertain the best method of curing it. The observations he has collected, although made in latitude 32° 30′ may not be inapplicable to a more northern one, as from the recorded experience of those who have seen the disease in various parts of the United States, he is induced to believe that it is essentially the same.

*Symptoms.*—The manner of the invasion of congestive fever varies. Some persons are taken down suddenly, without any premonition; others experience for a period, varying from one to four days, feelings of languor, lassitude, and the usual symptoms preceding other fevers; whilst in a third class, it will take the place of the cold stage of an intermittent, whether tertian, quotidian, or double tertian.

When the disease manifests itself, there is usually great depression of the muscular powers. The skin is of a pale ashy hue; it looses its natural elasticity, and if pinched up with the fingers, it will retain the form so given to it, being unable to regain its natural position. There is an icy coldness over the whole of the extremities, but some heat about the thorax and epigastrium; great restlessness—the patient tossing himself from one side of the bed to the other; when the intellect is not disturbed, the eyes have a peculiar expression of restless anxiety. There is great distress about the epigastric region, as if a weight were pressing on it. The respiration is hurried and oppressed, and the patient endeavors to assume the erect position in order to breathe freer. The pulse is small and frequent, and often very feeble. The tongue is paler than natural, covered with a thin white fur, and moist. There is usually nausea—sometimes vomiting. The thirst is intense. The bowels are generally loose, and the discharges are thin and of a dirty grayish aspect; if the liver be engorged, the dejections become reddish, and look like water in which butcher's meat has been washed. Such are usually the symptoms, when the encephalon does not bear the weight of the disease. But it sometimes happens that this organ is the first to suffer. In such cases the patient is seized at once with profound coma—the eyes are fixed—the limbs are rigid, and there is a total want of sensibility. Needles run into the skin provoke no evidence of being felt—the strongest rubefacients make no impression; the temperature of the surface is here also very unequal, the extremities being icy cold, whilst about the head and over the chest it is more elevated, The bowels are not so liable to be loose as in other cases, but if so, the discharges are involuntary. The pulse is oppressed, intermittent, and sometimes almost imperceptible; respiration is performed with difficulty.

Such conditions as the foregoing will continue from eight to thirty-six hours, and, unless reaction be established, will terminate in death. If reaction come on, by the force of the vital energies, it will usually do so from three to eight hours from the commencement of the attack. The reaction however, under such circumstances, is very imperfect. There is some elevation of temperature over the thoracic and abdominal regions. The extremities lose their icy coldness, but do not attain a natural heat. The skin is dry, or else covered with a profuse and exhausting perspiration—the pulse is fuller than it was, but still weak, compressible and frequent. There is, however, a great abatement of the restlessness and the functional distress generally. This feeble stage of reaction lasts until the next paroxysm comes on—usually, however, this is not so severe as the first, and such a state of things might throw the practitioner off his guard. The third paroxysm is more severe than the second, as will the fifth be more severe than the fourth. In this manner the disease is analogous to a double tertian intermittent. Cases are sometimes met with, however, in which the reaction is not only complete but very violent. This stage of reaction lasts from twenty-four to forty-eight hours, then to terminate in a collapse from which the patient never recovers by the unaided efforts of nature.

*Anatomical Characters.*—The morbid appearances found in persons who have died from congestive fever are various, but they agree in showing an abnormal accumulation of blood in some of the viscera. In the cavity of the head we sometimes find the arachnoid membrane to be injected, and the vessels of the brain distended and engorged with blood.

In the thorax, the lungs are nearly always engorged, and when cut into, exhibit portions of a dark red or brown color.

In the abdomen, the mucous membrane of the stomach and duodenum is usually injected—presenting that dark vascularity to which the term venous congestion is applied. The intestines sometimes partake of the same condition—sometimes do not differ from their natural appearances.

The liver is frequently gorged with blood, presenting a purple appearance; the density of its structure is sometimes increased, but more often it is softened.

The spleen is always affected—there is an increase of its size and consistence—it is easily torn—its normal structure is destroyed, the interior being pulpy and of a reddish black.

*Nature.*—The nature of congestive fever can best be investigated by an inquiry into its proximate cause. The lesions revealed by the scalpel are too evidently the result of the disease, to induce the pathologist to appeal to them to elucidate the morbid actions which produce its phenomena. We must clearly then study the disease itself from its inception to its termination, through all its phases, to arrive at its true nature. By doing so we are led to believe that the proximate cause of congestive fever lies in an irritated and perverted condition of the nervous system. The morbific cause, whatever it may be, which produces the disease, exerts its influence on the nervous system, by which innervation is no longer distributed in a healthy and regular manner. The suddenness of the attack in many cases, followed by profound coma, the stertorous breathing, the insensibility to all kinds of irritants, the diminished temperature, unequal in different parts of the body, the great restlessness, the periodicity of the attacks in cases where reaction comes on, all point to the important parts which the nervous system sustains in the disease. The nervous fluid which it supplies to the different organs of the body, to enable them to perform their functions, is no longer sent out in an equable and healthy manner; the harmony of actions in the human system is broken up, the heart and arteries, and capillary system, no longer perform their duties; the circulation of the blood becomes retarded, congestion of the internal organs takes place, secretions are arrested, or depraved; the capillaries no longer receiving their due supply of arterial blood, and innervation being perverted, calorification is altered and partially arrested—hence the diminished and unequal temperature.

*Causes.*—The predisposing causes of congestive fever are sufficiently obscure. They are unquestionably those which produce ordinary intermittent fever, but in a very concentrated state.

Congestive fever usually prevails in those localities where intermittent fever is rife; in fact, as has been observed above, a disease which commences as a well marked case of intermittent fever, will sometimes terminate in as equally well marked a case of congestive fever. Whenever this occurs it is in old persons, or those of an infirm constitution, or in habitual drunkards. We may well assume that congestive fever is nothing more than a malignant intermittent, with very little, or no power of reaction.

The exciting causes can be frequently traced to exposure to rain, when the body is heated and perspiring; more frequently it can be traced to the direct rays of the solar heat; hence in the south, laborers in the fields, and mechanics who work in the open air, are more frequently attacked than others. Often, too, it follows a simple remittent and intermittent by the abuse of evacuants; a powerful cathartic, which, by inducing large watery stools, prostrates the vital powers, converts those diseases into congestive fever. But this is really merely changing the quantity of disease.

*Treatment.*—Bloodletting has been highly recommended as a remedy of great value. It is supposed "to promote reaction by diminishing the load under which the heart and other vital organs are laboring." A little reflection however ought to convince us of the impropriety of this view. The morbid phenomena do not depend by any means on a mere physical accumulation of blood in the central organs. There is a previous cause which has produced the retreat of the blood from the periphery, and which continues to act. This is the change wrought in innervation by the lethiferous influence of the agent which has impressed the nervous system. The congestion is not owing to the amount of circulating fluid. The system is not laboring under too much blood, but an unequal circulation of it. Withdrawing any quantity will not prevent this unequal tendency to particular organs. If an animal be bled to death, autopsic examination will show, that notwithstanding the amount of blood lost, in fact in consequence of that very loss, congestion of the brain has ensued. Every one who has been extensively engaged in the practice of obstetrics, has had an opportunity of seeing symptoms of a marked determination of blood to the brain in women who have suffered from profuse uterine hemorrhage. Clearly then, the diminution of the quantity of the circulating fluid cannot prevent congestion. It seems to be independent of the amount of blood in the system. When it follows the loss of blood, it results from a nervous irritation which itself is produced from that loss; thus exhibiting how intimately the nervous and circulatory systems are connected, and how they act and react on each other. It is not pretended to be denied that plethora may induce a condition that will produce congestion, but the manner of action in such cases is obvious. We can now see that bloodletting, if it is to act in this purely physical manner, is useless. But it is worse than useless. It is pernicious. What have been the results of experiments made to test the accuracy of Dr. Mackintosh's views of the use of the lancet in the cold stage of an intermittent fever? They show that the plan has a great tendency to convert the intermittent into a remittent or continued fever—a result always to be deprecated. If this be the result in many cases of simple intermittent, what can we anticipate in the malignant form, known as congestive fever? where the powers of life are so nearly overwhelmed, that the patient looks as if he were in the collapsed stage of Asiatic cholera! It is true that in many cases of simple intermittent fever bloodletting has been practised in the cold stage with advantage. But such cases are easy of explanation. They arise in persons who are laboring under an inflammatory affection of some organ, and the congestion which ensues, as the result of deranged innervation during the cold stage, is accumulated upon this inflamed organ. In such cases, the increased hyperæmia might be productive of bad consequences—and in such cases the system will bear the loss of blood, in accordance with the general law which establishes a greater tolerance of bloodletting whenever an inflammatory diathesis prevails. Thus we often see persons of a weak habit of body bear the loss of a large amount of blood in some acute inflammation, which they could not do, even in a state of health.

In the present state of our knowledge it cannot for a moment be supposed that the phenomena of congestive fever are owing to the deleterious effects of the venous blood accumulated in the viscera. Researches have proved that venous blood enables the nervous system and the various organs of the body to preserve their vitality much longer than when the blood has been entirely withdrawn, and therefore it cannot be poisonous to the functions of innervation, &c. (*Influence of Physical Agents on Life,* by Dr. W. F. Edwards). In truth, if the pathology of the disease be a lesion of innervation, the nervous system being in a state of sedation and irritation, it would be as rational to bleed in the oppressed stage of it, as it would be to bleed in a case of violent concussion of the brain before reaction came on. Bloodletting by diminishing the amount of the circulating fluid lessens the probabilities of reaction, and thus increases the danger. But after reaction is established, it may be necessary to withdraw blood under circumstances and in the manner presently to be described.

The indication in the oppressed stage of congestive fever is, to rouse the nervous system so as to enable it to perform its functions. Remembering that although there is depression of the vital manifestations, there is also an irritated condition, in which the nervous fluid is unequalised and misdirected, we must resort to those remedies which are capable both of stimulating and tranquillizing the nervous system. Steadily bearing in mind however the proneness of congestion to run into inflammation, we should avoid those stimulants which would be apt to create an inflammation after reaction came on. Fortunately however our art has afforded us remedies well adapted to this disease—and these are quinine and opium. The utility of opium in allaying the irritability of the nervous system is well known. Its use in cutting short the cold stage of an intermittent is beyond doubt—quinine has powers of exciting the nervous system with but little danger of lighting up inflammation. In addition to these, it is proper to use the cold dash, frictions, &c., in the manner now to be described.

If called to a case during the stage of depression, it was the custom of the writer to give about ten grains of the sulphate of quinine with a grain or two of opium, to be repeated every two hours until reaction came on. If the stomach was too irritable to bear this, it was used in injection, taking care that the vehicle should be so small in quantity that it would be retained; at the same time endeavors were made to restrain the irritability of the stomach by means of small pieces of ice (when it could be obtained), sinapisms to the epigastrium, the effervescing draught, &c.

In addition to the method of reaching the nervous system by remedies administered internally, its peripheric extremities so profusely spread over the cutaneous tissue were appealed to. With this view the cold dash was used. This remedy might seem in such cases less consonant with reason than bloodletting. To dash cold water over a person already so cold that his skin feels like ice might appear not rational. But when we remember how great an excitant to the nervous system the cold *doûche* is, its efficacy will no longer be doubted. Hence if one falls into a state of syncope, the first thing that suggests itself to the bystander's mind, is to throw cold water into the face. Hence too, when a patient is laboring under narcotism, the same remedy is universally used.

The method of applying the cold dash is to place the patient on the floor and having a tub of cold water at hand, and taking a pitcher full, to pour a stream over him, from an eminence of about six feet, taking care to move the pitcher so that the fall of water shall strike the head, thorax and abdomen, in rapid succession. Having continued this until signs of reaction are manifested, he is to be removed into bed enveloped in a blanket, and frictions are to be applied assiduously all over the body. This may be done with the bare hand, or with a piece of flannel or the flesh brush dipped into spirits of turpentine.

Under this treatment reaction is often established; if the stage of depression come on again, the same remedies are again to be used.

When reaction is established we are to guard against inflammation. This does not often occur. If, however, the disease has been ushered in with diarrhœa, and there is tenderness on pressure over the abdomen, the reaction is apt to be violent, and if not checked will terminate in a fatal collapse. Here is apt to arise a case complicated with enteritis. Under such circumstances we must rely on local blood-letting, emollient fomentations, and small doses of calomel, combined with opium and ipecac., administered at intervals of two or three hours. To this treatment the phlogosis will generally yield. Sometimes in persons of a plethoric habit, the brain seems to suffer after reaction. Here, the application of the cupping glass to the back of the neck, cold applications to the head, sinapisms to the feet, and mercurial cathartics, will remove the symptoms. Having subdued the local irritation we resort to quinine in sufficiently large doses to prevent the recurrence of the paroxysm. It may sometimes happen that we have reason to suspect local irritation, and yet dread the return of another paroxysm which may prove fatal. This happens in old persons, and in those of an enfeebled constitution. Are we to subdue the local irritation first and wait to give quinine? or to give the quinine before the local irritation is quelled? The writer's practice was, never to run the risk of a second paroxysm under such circumstances—as soon as the first one was off he gave 10 grains of quinine, using at the same time extensive counter-irritation over the seat of the suffering organ. With this view a sinapism was applied until a deep redness of the skin was produced, and then a vesicatory was applied over the part. The quinine used in this manner never produced any bad consequences, whilst it averted the danger of a second attack.

In the large majority of cases, however, the period of re-action is not marked with any inflammatory condition. Our attention must be directed to keeping off the return of the paroxysm. We of course then use quinine fearlessly, and it is well to commence as soon as the stage of reaction has come on; because quinine is not a diffusible stimulant, and requires some time before its effects on the system are felt—at least it certainly requires that it should have been introduced into the system some hours before its anti-periodic effects are developed. In cases, therefore, which are not complicated with diarrhœa, we omit the opium given with the quinine in the stage of depression, and continue the quinine as before.

If diarrhœa should be present, which is often occasioned by irritation in the ganglionic system of nerves, whereby the balance between the exhalents and absorbents is lost, and a large quantity of fluid is poured into the intestines, we must continue the opium. When the reverse happens, and we have costiveness, it is necessary to keep the bowels open with mild cathartics and enemata. After the period of the anticipated return of the paroxysm is passed, it is proper to allow the patient generous but not stimulating diet, and vegetable tonics; as his health suffers from the effects of the disease for some time afterwards.

April, 1843.

